# Comparison of the efficacy of the cervex brush and the extended-tip wooden spatula with conventional cytology: A longitudinal study

**DOI:** 10.4103/1742-6413.45192

**Published:** 2009-01-19

**Authors:** Caroline J Whitaker, Elaine C Stamp, William Young, Lesley A Greenwood

**Affiliations:** Quality Assurance Reference Centre, Unit 9 Kingfisher Way, Sliverlink Business Park, Wallsend, Tyne and Wear, NE28 9ND, UK; Institute of Health and Society, Newcastle University, 21 Claremont Place, Newcastle upon Tyne, NE2 4AA, UK; 1 Department of Pathology, Hull Royal Infirmary, Anlaby Road, Hull, East Yorkshire, HU3 2JZ, UK; 2 Cervical Screening Training, Unit 9 Kingfisher Way, Sliverlink Business Park, Wallsend, Tyne and Wear, NE28 9ND, UK

**Keywords:** Cervex brush, conventional cytology, extended-tip wooden spatula

## Abstract

**Background:**

Within the United Kingdom, the change from conventional to liquid based cytology (LBC) has brought with it the universal introduction of broom style samplers, as represented by the Cervex sampler. The aim of this study was to assess whether or not there were benefits associated with a change from wooden spatulae to broom style samplers for those countries where conversion to LBC might not be readily available or is not fully supported.

**Methods:**

A longitudinal study was designed to compare the performance of Cervex brushes and extended-tip wooden spatulae as sampling devices for conventionally prepared cervical smears. General Practices serving the population of Hull and East Yorkshire (UK) were provided with Cervex brushes for a period of nine months to routinely collect cervical smears. The results of 66,931 cervical smear tests were compared between those practices that were using extended-tip wooden spatulae before the trial and then returned to their use afterwards, and those who were previously using Cervex samplers and continued to use them throughout. Analyses comparing both specimen inadequacy, as recorded on the standard cervical screening request form (HMR101), and also the presence of identified transformation zone (TZ) elements in smears, both indicated significant advantages associated with the Cervex brush.

**Results:**

Inadequate smears decreased from 5.96% with extended-tip spatulae to 4.77% with Cervex brushes (p<0.001) and increased back to 7.34% when practices reverted to extended-tip spatulae after nine months. Under the same conditions, the proportion of smears containing identified TZ elements increased from 50.52% to 54.75% (p<0.001), before reverting to 45.47% (p<0.001). In contrast, for a control group of practices using the Cervex brush throughout, inadequate smears decreased in all phases of the study, with no significant variation in TZ sampling rates.

**Conclusions:**

Using the Cervex brush with conventional cytology significantly decreases inadequate smears and increases TZ sampling when compared to the extended-tip spatula and can offer improved cervical screening in countries unable or unwilling to convert to LBC.

## BACKGROUND

In England, the NHS Cervical Screening Programme (NHSCSP) is currently undergoing the largest change to cervical smear taking, processing and screening since the introduction of the Programme in 1988. By 2008, all laboratories processing smears for the NHSCSP were scheduled to have undergone conversion from conventional cytology to liquid based cytology (LBC). It is anticipated that the introduction of LBC in the NHSCSP will decrease inadequate smears and shorten both screening and reporting times.[1] These anticipated changes are believed to be mainly due to the alteration in smear processing and screening. However, a large part of the planned change from conventional cytology to LBC also involves an alteration in smear taking device from the traditional extended-tip wooden spatula to the Rovers^®^ Cervex-Brush^®^ (Cervex brush), along with a re-training of all smear takers.

**Figure 1:**
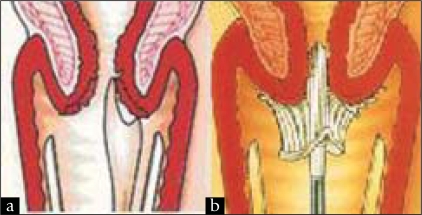
Diagrams showing the use of the (a) extended-tip spatula and (b) Cervex brush to take cervical smears (Images reproduced with permission from Rovers Medical Devices)

Unlike the traditional wooden extended-tip spatula, which is rigid and shaped to follow the contours of a ‘typical’ cervix, the Cervex brush was designed with flexible plastic bristles which can follow the individual shape of the woman's endocervix, ectocervix and transformation zone (TZ). As shown in [Figure 1], this should result in more effective sampling of the area of the cervix where most abnormal cells develop.

Smear taking devices which collect endocervical cells effectively have been shown to detect a higher proportion of abnormal cytology than those that do not[2] and the presence of these endocervical cells has also been demonstrated to be a valid and convenient surrogate for the ability to detect dyskaryosis.[2] Smears lacking TZ cells identify less squamous abnormalities than those with TZ sampling.[3] In particular, smears which lack endocervical cells do not allow screening for glandular atypia.[2]

As early as 1989, shortly after its introduction, there was a suggestion that the use of the Cervex brush instead of wooden spatulae both decreased the rate of ‘unsatisfactory’ smears and increased the proportion of smears which contained endocervical cells.[4] However, since then, several studies have been published investigating these same factors, with highly conflicting results.

In 1996, Dey *et al*,[5] published the results of a trial which directly compared inadequate rates from smears taken in primary care with either the extended-tip spatula or the Cervex brush. This study showed that there was no significant difference in inadequate rates from smears taken with the Cervex brush compared to smears taken with the extended-tip spatula, although, it is worth noting that in this study, training for using the Cervex brush was not mandatory. Meta-analysis of a number of papers which compared smears taken with the Cervex brush to those taken with a spatula has also showed no significant difference over all in inadequacy rates,[2] however, there was a large range of variability in the results of the studies analysed. In contrast, a study by Williamson *et al*,[6] of smears taken using one of three cervical sampling devices, showed that there was a significant higher proportion of ‘good quality’ smears taken with the Cervex brush than with the extended-tip spatula.

It was a combination of the conflicting results of these published studies, and the impending change to LBC, coupled with the knowledge that appropriate smear taker training can have a beneficial effect on inadequate smear rates,[7] which led to this study. The aim of which was to analyse the effects of changing cervical sampling device, with additional smear taker training, on inadequate smears and TZ sampling using conventional cytology.

## MATERIALS AND METHODS

Medical practices within the city of Kingston Upon Hull and the surrounding rural area of East Yorkshire were selected for inclusion in this trial because their relative geographical isolation restricts cross boundary flow and ensures that all smears are sent to the same laboratory, at the Hull Royal Infirmary, for reporting. The use of a single central laboratory in this study ensured a high degree of consistency in the interpretation and classification of smears. In addition, the laboratory information system in use at Hull Royal Infirmary allows recording and retrieval of additional smear information, including smear taker identity and the presence or absence of TZ elements.

Reporting of smears at Hull Royal Infirmary adheres closely to both NHSCSP[8] and regional guidance on the criteria for specimen adequacy, with the recognised criteria for inadequacy being one of the following: insufficient cellular material present, separated superficial cells in a pattern suggesting vaginal sampling only, inadequate fixation causing extensive drying artefact, obscured by blood and/or inflammatory exudates, too thickly spread for assessment, the cervix was not fully visualised or no TZ cells identified following a previous glandular abnormality. In particular, the presence of endocervical cells is not considered an essential requirement for the classification of a smear as adequate.

**Figure 2:**
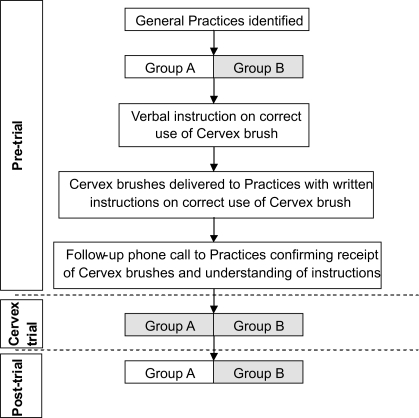
Flowchart showing stages involved in the study. Shaded boxes indicate practices using Cervex brush to take cervical smears

The General Practices enrolled into the study were divided into two sets based on their existing method of collecting cervical smears [Figure 2]. One group included those practices using the extended-tip spatula who, following a period of using the Cervex brush, would eventually revert to using their original device. The second (control) group was made up of practices who had already converted to using Cervex brushes and who would continue to use them throughout the trial and the ensuing period.

Funding was secured from Cervical Screening Training to provide all General Practices within this area with nine months supply of Cervex brushes.

Before the Cervex brushes were sent to practices, verbal instruction in their correct use was given during one session of the conference organised 3-yearly for smear taker update training, as recommended by the NHSCSP. The instruction lasted approximately 15 minutes and comprised of a presentation and a demonstration of the correct use of the Cervex brush. In addition, full printed instructions were included with the packs of Cervex brushes distributed to each participating practice. In order to confirm that the packs of brushes had been received and that the associated instructions had been read and fully understood, each practice was contacted via phone by a smear taker trainer prior to commencement of the trial. This same protocol was carried out with all General Practices, independent of whether or not they had previously used, or were currently using, the Cervex brush [Figure 2].

Smear takers were instructed to visualise the cervix, position the tip of the Cervex brush in the os and complete five 360° sweeps in a clockwise direction to sample the whole of the TZ.[9] Cellular smears were transferred from the Cervex brush onto slides, as in conventional smear taking, and the resulting smears were processed and screened by the cytology laboratory at Hull Royal Infirmary in the usual way for conventional smears.

The laboratory was not informed of which practices were routinely using the Cervex brush prior to the study and which practices had changed cervical sampling device. At the end of the specified nine months, General Practices reverted to using their original cervical sampling device [Figure 2].

The laboratory database was used to obtain details of all individual smears processed during the three time periods used in this study – December 2003 to July 2004 (pre-trial baseline analysis), December 2004 to August 2005 (trial period of exclusive Cervex brush use) and August 2005 to June 2006 (post-trial period with practices reverting to their original sampling device). In order to ensure consistency of laboratory reporting throughout the study, the staff screening and reporting the smears did not undertake any re-training in advance of LBC implementation until all phases were completed.

The data collected included the date the smear was received by the laboratory, cytological opinion on the smear relating to adequacy and any abnormalities detected using the British Society for Clinical Cytology coding definitions,[8] along with a unique code for each smear taking location. This unique code was then used to assign each smear to a particular group of General Practices, as shown in Table 1.

**Table 1:** Cervical sampling device used in each of the three periods of the study

*Date of smear*	*Group A*	*Group B*
Dec 2003 - Jul 2004 (Pre-trial)	Extended-tip spatula	Cervex brush
Dec 2004 – Aug 2005 (Cervex trial)	Cervex brush	Cervex brush
Aug 2005 – Jun 2006 (Post-trial)	Extended-tip spatula	Cervex brush

In total, 66,931 smears were included in the analysis from 95 individual General Practices. In some practices it was not possible to confirm that a particular sampling device had been used consistently throughout the pre-trial period and, where there was any possibility that a mixture of spatulae and brushes may have been used, their results were not included in the analysis.

For the purposes of analysis those practices using the extended-tip spatula prior to the trial were classed as Group A whilst those already using the Cervex brushes were classed as Group B. The percentages of negative, inadequate and abnormal smears during the three time periods were calculated for each General Practice. Using the paired Student's T-test, practices in Group A were compared during the Pre-trial and Cervex trial period, and the Cervex trial and Post-trial period for statistically significant differences in the percentage of negative, inadequate and abnormal smears. The same analysis was carried out for practices in Group B as a control. The three major results categories (negative, inadequate and abnormal) each contained sub-categories which were also analysed in the same way, when smear numbers were large enough to allow statistical analysis.

## RESULTS

A complete set of data was collected from 95 General Practices which were split into two groups dependent on their use of the Cervex brush prior to the start of this study [Table 1]. There were 62 practices which routinely used the extended-tip spatula prior to the study (Group A: Table 2). The remaining 33 practices were confirmed to use the Cervex brush prior to, and throughout, this study (Group B). Table 2 also shows the average age of the women whose smears were included in each of the time periods for each group of General Practices.

**Table 2:** Table showing the number of smears included in each period of the study and the average age of women whose smears were included in each period

*Time period*	*Group A*		*Group B*	
		
	*No. of smears*	*Average age (years)*	*No. of smears*	*Average age (years)*
Pre-trial	14148	40.8	9898	41.3
Cervex trial	15126	41.0	9801	41.6
Post-trial	11488	41.8	6480	42.6
Total	62 practices		33 practices	

The results from 66,931 cervical smears (Group A – 40,752 smears, Group B – 26,179 smears) were collected and analysed as described. Smears were assigned to one of three time periods; Pre-trial, Cervex trial or Post-trial, depending on the date the smear was taken. The summary and analysis of these results, along with a detailed sub-division of the inadequate, negative and abnormal reports into the categories used at Hull Royal Infirmary is shown in Table 3.

**Table 3:** Table showing the number of smears included in each result category and each period of the study Asterisk indicates a significant difference between that result and the previous study period

*Category code and definition*	*Group A*						*Group B*					
		
	*Pre-trial*		*Cervex trial*		*Post-trial*		*Pre-trial*		*Cervex trial*		*Post-trial*	
						
	*No.*	%	*No.*	%	*No.*	%	*No.*	%	*No.*	%	*No.*	%
1 Inadequate	1	0.01	1	0.01	3	0.03	3	0.03	0	0.00	1	0.02
11 Inadequate	0	0.00	0	0.00	73	0.64	2	0.02	0	0.00	15	0.23
12 Insufficient cellular material	564	3.99	471	3.11	527	4.59	345	3.49	298	3.04	133	2.05
13 Separated superficial cells suggesting vaginal sample	3	0.02	2	0.01	0	0.00	1	0.01	3	0.03	2	0.03
14 Inadequate fixation	7	0.05	7	0.05	4	0.03	2	0.02	1	0.01	0	0.00
15 Obscured	245	1.73	226	1.49	220	1.92	171	1.73	130	1.33	89	1.37
16 Too thickly spread	1	0.01	3	0.02	5	0.04	2	0.02	0	0.00	0	0.00
17 Mainly endocervical	10	0.07	6	0.04	2	0.02	7	0.07	10	0.10	4	0.06
18 Cervix not fully visualised	10	0.07	6	0.04	8	0.07	5	0.05	5	0.05	1	0.02
19 NoTZ cells identified	2	0.01	0	0.00	1	0.01	0	0.00	0	0.00	0	0.00
Total inadequate	843	5.96	722	4.77	843	7.34	538	5.44	447	4.56	245	3.78
2 Negative	3478	24.60	3683	24.35	2678	23.31	2435	24.60	2586	26.39	1913	29.52
21 NegativeTZ cells not seen	2067	14.62	1689	11.17	2094	18.23	1247	12.60	1077	10.99	234	3.61
22 NegativeTZ cells present	7142	50.62	8282	54.75	5224	45.47	5245	52.99	5187	52.92	3664	56.54
Total negative	12687	89.74	13654	90.27	9996	87.01	8927	90.19	8850	90.30	5811	89.68
8 Borderline	385	2.72	450	2.98	414	3.60	258	2.61	315	3.21	279	4.31
3 Mild	90	0.64	110	0.73	88	0.77	51	0.52	67	0.68	56	0.86
7 Moderate	65	0.46	93	0.61	69	0.60	69	0.70	59	0.60	50	0.77
4 Severe	62	0.44	83	0.55	70	0.61	50	0.51	58	0.59	36	0.56
5 Severe/?invasive	3	0.02	6	0.04	6	0.05	3	0.03	1	0.01	0	0.00
6 ?glandular	3	0.02	8	0.05	2	0.02	2	0.02	4	0.04	3	0.05
Total abnormal	608	4.30	750	4.96	649	5.65	433	4.37	504	5.14	424	6.54
Total	14138	100	15126	100	11488	100	9898	100	9801	100	6480	100

?invasive = suspicious for invasion; ?glandular = suspicious for glandular

## DISCUSSION

Although the practices during the trial were randomly distributed throughout Hull and East Yorkshire there were some concerns that significant variation in the age profile between practices or during the study might act as a confounding factor – for example practices submitting smears from an increased percentage of postmenopausal women might see an increased number of smears reported as inadequate due to low cellularity. It was noted that the average age of the women in both groups increased over the course of the study [Table 2] and it is considered that this may relate to the general decrease in cervical screening coverage in younger women in recent years.[10] However, although the Group B practices had a consistently greater average age than Group A practices, the maximum difference between the two groups was less than a year, which was considered unlikely to account for any significant variation in reporting trends.

In assessing and comparing the effectiveness of two cervical sampling methods, the two fundamental areas to be considered are their influence upon specimen adequacy and, closely related to this, how effectively they support the detection of cytological abnormalities.

**Figure 3:**
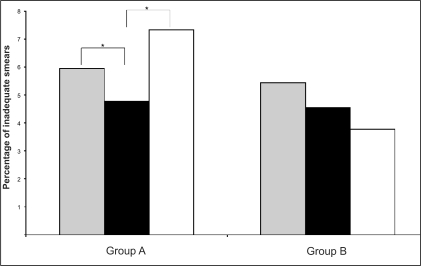
Percentage of inadequate smears in each of the three study periods – Pre-trial (grey bars), Cervex trial (black bars) and Post-trial (white bars) – for the two groups of General Practices (Group A and B). Asterisk indicates a significant difference between the two time periods indicated

One unequivocal finding of this study was the significant decrease in inadequate smears when the Cervex brush is used as opposed to the extended-tip spatula [Table 3, Figure 3]. Whilst this improvement may have been due in part to the element of re-training incorporated in the study the evidence suggests that the major contribution to the reduction in inadequate smears stems from the choice of sampling device.

The use of the control General Practices (Group B), who continued to use the same sampling device throughout all phases of the study, allowed monitoring of the effect that the additional smear taker training achieved in reducing inadequate smears. Practices within the control group received the same smear taker training as Group A practices, despite already using the Cervex brush prior to the trial. Hence, the significant (*p*<0.001) decrease in the percentage of inadequate smears for Group A practices upon switching to the Cervex brush could not be entirely attributed to additional smear taker training received as part of this study, since the decrease seen in the similarly re-trained control group (B) showed a much less significant (*p*=0.023) decrease for the same period and throughout the study. However, in order to specifically investigate the effect of smear taker training on inadequate smears, a further control group of practices, which received no additional smear taker training, would need to be included in any future studies. In addition, the subsequent large increase in inadequate smears which occurred when Group A changed from using the Cervex brush back to using the extended-tip spatula, cannot be attributed to a ‘fall-off’ following smear taker training, since Group B practices did not show this same effect. In fact, inadequate smears for Group B practices continued to decrease over the entire study period [Table 3, Figure 3], which may demonstrate the continued beneficial effect of the additional smear taker training received as part of this study.

It has previously been demonstrated that changing from one sampling device to another which requires more pressure to obtain an adequate smear, initially results in a large increase in inadequate smear rates.[11] However, in this study where there was a change to a device (Cervex brush) which requires less pressure to obtain an adequate smear than with the previous device (extended-tip spatula), there was an overall decrease in inadequate smears. This reduction occurred as soon as the device was changed, with no measurable learning curve effect detected (data not shown). This is in contrast with the study by Cross *et al*,[11] in which it took up to ten months to return inadequate rates to previous levels before the sampling device change. When the practices in Group A reverted to the use of wooden spatulae at the end of the trial period, they were effectively reproducing the change to a device requiring more pressure and this was reflected in an increase in the inadequate smears and, in this case, the increase was entirely consistent with the previously described learning curve effect.

It is clear that a decrease in inadequate smears is of benefit to several aspects of any cervical screening programme. Firstly, the woman does not have to attend for a repeat smear which prevents the associated anxiety and also does not take up valuable Primary Care time. Secondly, a decrease in inadequate smears reduces laboratory workload with potential knock-on benefits of reduced cost and improved reporting times in general. Finally, since it is NHSCSP guidance[12] that all women are referred to colposcopy following three inadequate smears, decreasing the overall inadequate rate would hopefully decrease the number of these women who are referred to colposcopy. This would consequently decrease anxiety and over-treatment, as well as freeing-up valuable colposcopy time for women with actual cervical disease.

**Figure 4:**
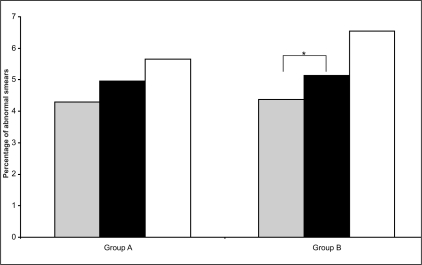
Overall abnormal smear rates as a percentage of total smears in each of the three study periods – Pre-trial (grey bars), Cervex trial (black bars) and Post-trial (white bars) – for the two groups of General Practices (Group A and B). Asterisk indicates a significant difference between the two time periods indicated

Although the beneficial reduction in inadequate smears associated with the change in sampling devices is immediately apparent, the potential advantages associated with increased disease detection are less obvious. [Figure 4] shows the effect the different sampling regimes had on the overall percentage of abnormal smears detected over the course of this study. The percentage of abnormal cytology detected from both groups of General Practices increased over all three time periods: from 4.30% to 4.96% and 5.65% for Group A practices and from 4.37% to 5.14% and 6.54% for Group B practices. There was a significant (*p*=0.030) increase in abnormal smear rates for Group B practices between the Pre-trial and Cervex trial period, however, none of the other increases reached significance.

**Figure 5:**
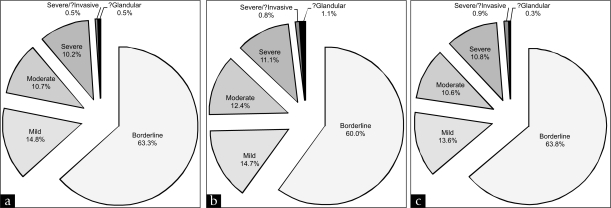
Proportion of each reporting category of abnormal smears detected in (a) Pre-trial, (b) Cervex trial and (c) Post-trial time periods in Group A practices

Table 3 shows the six categories used for abnormal smears, which are ordered from the least severe to the most severe level of disease. The proportion of smears in each category decreases with increasing severity [Figure 5], with over half of smears (62.4%) being reported as the least severe abnormal category: borderline. In [Figure 5a] the data are shown for the Pre-trial abnormal smears, in [Figure 5b] for the Cervex trial and in [Figure 5c] for the Post-trial.

Although the inclusion of almost 67,000 results makes this one of the largest studies which has been conducted to use a common training regime and to directly compare the efficacy of sampling devices, the total number of unequivocally abnormal smears is still relatively low. Since the vast majority of cervical smears routinely taken in General Practice will have negative cytology, over 89% in this study, it is clearly necessary to analyse the results of a very large number of smears before any reliable conclusions can be drawn regarding any directly measurable changes in the rates of unequivocally abnormal smears detected. It is particularly hard to recognise any trends in the most severe disease categories since these constitute such a small proportion of total smears. For example, smears showing evidence of possible glandular neoplasia constituted only 0.033% (22 smears) of the total smears in this study.

**Figure 6:**
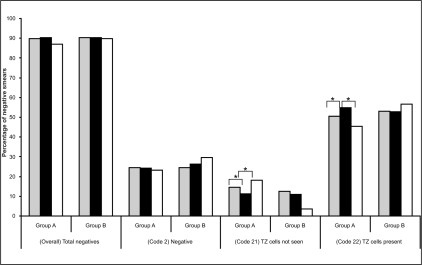
Percentage of overall (total negative smears) and subcategories (the negative smear code 2, code 21 and code 22) in each of the three study periods – Pre-trial (grey bars), Cervex trial (black bars) and Post-trial (white bars) – for the two groups of General Practices (Group A and B). Asterisk indicates a significant difference between the two time periods indicated

However the findings do provide some evidence that, in the longer term, the number of cervical abnormalities detected might be expected to be raised by virtue of the change to the Cervex brush. This evidence emerges from further consideration of the smears recorded as being negative, but subdivided according to whether TZ elements were identified or not [Table 3, Figure 6]. Once again the evidence suggests that changes are not simply due to the additional smear taker training which was carried out between the first two time periods. In Group A practices, the significant increase in negative smears which were classified as ‘TZ cells seen’ when using the Cervex brush indicates that the Cervex brush gives increased sampling of the TZ, when compared to the extended-tip spatula. This may be expected since the bristles of the Cervex brush can be inserted more easily into the os to allow increased sampling of the endocervical canal [Figure 1]. However, the extended-tip spatula may not easily sample the endocervical area, especially if the woman has a small os due to being nulliparous, post-menopausal or following treatment.

For Group A practices, there was a significant (*p*<0.001) decrease in the percentage of negative smears which were reported as ‘TZ cells not seen’ (code 21), upon the implementation using of the Cervex brush, from 14.62% to 11.17%. This effect was reversed when these practices reverted to using the extended-tip spatula, with smears coded as ‘TZ cells not seen’ increasing from 11.17% to 18.23% (*p*<0.001). This was echoed in the number of negative smears recorded as ‘TZ cells present’ (code 22) which increased significantly (*p*<0.001) with the use of the Cervex brush from 50.52% to 54.75% and decreased significantly (*p*<0.001) to 45.47% with the reinstatement of the extended-tip spatula. However, this was not observed with Group B practices, which showed no significant changes in any of the two negative sub-categories throughout the study [Figure 6].

This clearly demonstrates that use of the Cervex brush with conventional cytology gives increased sampling of the TZ, compared to the extended-tip spatula [Table 3, Figure 6] and supports the evidence provided by other papers which have used the review and analysis of a number of much smaller studies to reach similar conclusions.[2,13,14]

The increase in abnormal smears over the entire study was similar for both groups of practices, with the only significant increase occurring in the control Group B practices between the Pre-trial and Cervex trial periods. These results would appear to indicate that changing cervical sampling device from the extended-tip spatula to the Cervex brush has little or no directly measurable effect on abnormal smears. However, this appears to contradict the significant increase in TZ sampling, demonstrated here and resulting from the same change in cervical sampling device, since the TZ is precisely the area where dykaryosis and other cervical abnormalities tend to develop.[15] It may be that effective TZ sampling affects the pick-up rates of some categories of abnormalities more than others. Another possibility is that the abnormalities detected using the Cervex brush may be a more accurate representation of true disease, leading to a closer correlation between cytology and histology. However, these possibilities would have to be explored in a further study, perhaps by undertaking a slide review along with a comparison of smear and colposcopy results.

An indication of the variable effect of TZ sampling on abnormal categories is currently emerging from the NHSCSP as laboratories convert to LBC. There already is anecdotal evidence from laboratories which have recently converted from conventional cytology to LBC, along with one preliminary publication[16] that there has been an increase in glandular smears and an improvement in glandular abnormality detection. This is also reflected in the annual statistical return (KC61) produced by the NHSCSP[17] which is based upon cervical reporting data submitted by every screening laboratory using a standard form to summarise all reports issued within the year. Percentages of smears reported as potential glandular abnormalities across England as a whole have been falling since 1997/1998, when they constituted 0.0936% of all smears, to a low of 0.0522% in 2004/2005. However, for the first time in nine years, there was a small *increase* in glandular results in 2005/2006, to 0.0536%^©^,[17] which coincided with the beginning of the large scale implementation of LBC across England. Data from 2006/2007 (the most recent data available at the time of publication), in which many more laboratories across England converted to LBC, showed another increase in glandular abnormalities detected, to 0.0563%^©^.[17] Data currently being gathered, but as yet unpublished, from laboratories within a large region of England categorised as the North East, Yorkshire and The Humber indicates that, in laboratories that have converted to LBC, the percentage of smears showing evidence of TZ sampling has risen by between 10% and 12% and that the detection rates for glandular abnormality (confirmed by biopsy) have also been significantly increased. Since the majority of General Practices switched from using the spatula to the Cervex brush with the introduction of LBC, it may be that the change from the extended-tip spatula to the Cervex brush could, at least in part, reflect some of the increased detection of glandular neoplasia following conversion from conventional cytology to LBC.[18,19]

The underlying principle, that the clear increase in a commonly seen component of cervical smears (TZ elements) can act as an indirect indicator of a future and more subtle change in a relatively rare finding (high grade cervical intraepithelial neoplasia or invasive carcinoma) is further supported by the observations of Young[20] and Narine and Young[21] which demonstrate the statistically significant link between increasing TZ sampling rates and increased detection of cervical abnormality.

## CONCLUSIONS

Although the NHSCSP within England is already in the process of converting all laboratories to LBC, which includes changing cervical sampling device from the previously recommended extended-tip spatula to the Cervex brush, there are still many countries which are routinely using a spatula to take cervical smears and which are extremely unlikely to convert to LBC due to the large costs involved. In this study we have clearly demonstrated that changing from using the extended-tip spatula to the Cervex brush would significantly decrease inadequate smears and increase TZ sampling, which are crucial factors for improving any cervical screening programme. Although the Cervex brush is more expensive than the extended-tip spatula, Dey *et al*,[5] calculated that this difference in price between the two sampling devices can be covered by a 1.5% decrease in inadequate smears and hence their associated costs. The decease in inadequate smears observed in this study is within this order.

Hence it is suggested that, in order to decrease inadequate smears, increase TZ sampling and, as a consequence, potentially improve the detection of cervical neoplasia, conventional cervical smears should be taken with a Cervex brush as opposed to an extended-tip spatula.

## COMPETING INTERESTS

None to declare.

## AUTHORS' CONTRIBUTIONS

CJW performed part of the data analysis and drafted the manuscript. ECS participated in the design of the study, collated the raw data and performed part of the data analysis. WY participated in the study design and acquired the raw data. LAG conceived of the study, and participated in its design and coordination. All authors read and approved the final manuscript.

## ETHICS STATEMENT BY ALL AUTHORS

This study was conducted with approval from Institutional Review Board (IRB) (or its equivalent) of all the institutions associated with this study. Authors take responsibility to maintain relevant documentation in this respect.
